# Rejuvenating nerve cells in adults

**DOI:** 10.18632/aging.100574

**Published:** 2013-07-11

**Authors:** Hui Chiu, Chieh Chang

**Affiliations:** ^1^ Division of Developmental Biology, Cincinnati Children's Hospital Research Foundation, Cincinnati, Ohio 45229; ^2^ Division of Biology, California Institute of Technology, Pasadena, CA 91125

Like mammalian neurons, *C. elegans* neurons lose regeneration ability as they age, but it is not known why. *C. elegans* is a soil worm with its brain wiring diagram being mapped entirely - every connection between every nerve cell. Forty percent of genes identified in the worm genome have a counterpart in humans. Genes that allow neurons to connect with each other to form functional neuronal circuits and to regenerate themselves after injury are highly similar between worms and humans. Thus, what we learn in worms will likely be relevant to the development and regeneration of the human nervous system. The *let-7* microRNA and its target, the LIN-41 tripartite motif protein, were recently shown to function as neuronal timers in worms to time the decline of the ability of neurons to regenerate as they age [1]. The progressive increase of *let-7* and the progressive decrease of *lin-41* in neurons provide intrinsic timing mechanism [1].

**Figure 1:**
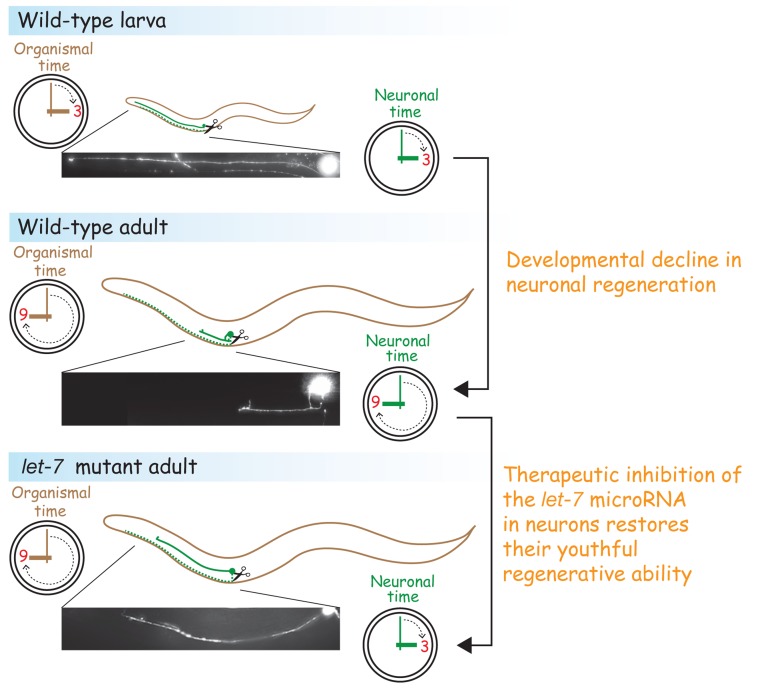
*let-7* mutations turn back the clock on regeneration in adult neurons Like many tissues in the body, the ability of neurons to regenerate new axons changes throughout the lifecycle, typically diminishing with age. Inhibiting *let-7*, or alternatively, increasing the level of its reciprocal inhibitor, *lin-41*, in adult neurons restored the regeneration capabilities of the larval axons. The dashed green lines indicate the disconnected axons in degeneration. The solid green lines are regenerating axons.

These discoveries have important implications in treating brain and spinal cord injury or neuro-degenerative diseases as they show that it may be possible to improve the ability of neurons in the adult brain to regenerate after injury through therapeutic inhibition of the *let-7* microRNA, and thereby restore their youthful regenerative capacity. MicroRNA does not encode a protein but rather a small RNA that imperfectly base-pairs to complementary sequences at 3' untranslated region (3'UTR) of target mRNAs in order to block gene expression [2, 3]. Approximately one third of the *C. elegans* microRNAs are evolutionarily conserved, implicating a central role for microRNAs in animals. The expression of microRNAs is either spatially restricted or temporally regulated in the nervous system. The spatially restricted expression of *lsy-6*, *mir-273*, and *mir-71* controls left-right asymmetry in neuronal development [4-6] while the temporally regulated expression of *lin-4* and *let-7* controls timing of neuronal connectivity and developmental decline in neuronal regeneration [1, 7]. The *let-7* microRNA represses the expression of *lin-41* to inhibit anterior ventral microtubule (AVM) axon regeneration in older neurons and the effect of *let-7* and *lin-41* in regulating AVM axon regeneration is mediated through the LIN-29 transcription factor [1]. Since *let-7* and *lin-41* genes are broadly expressed in different types of neurons [1], their roles in neuronal regeneration may be widespread. In addition to *let-7*, many microRNAs are also expressed in postmitotic neurons, raising the possibility that other microRNAs could also contribute to developmental decline in neuronal regeneration [1]. In *C. elegans*, many aged neurons display a further decline in axon regeneration. In aged AVM neurons, a reduced *let-7* remains able to enhance axon regeneration so it is likely that *let-7* continues to contribute to the further decline in axon regeneration in aged neurons [1]. This result also argues for a more direct role for *let-7* in axon regeneration, rather than it all being a simple delay in terminal differentiation of the neuron. Like *C. elegans* neurons, mammalian neurons also suffer from the age-related decline in axon regeneration. The idea of slowing down neuronal aging to promote axon regeneration after injury is an appealing possibility. Our results suggest that one way to promote axon regeneration is to turn back the clock in old neurons via manipulation of the neuronal timing microRNA, such as *let-7* [8]. Given that the *let-7* microRNA sequence and its late-onset expression are highly conserved across animal phylogeny, general rules of *let-7* microRNA governing neuronal regeneration in *C. elegans* are likely to be applicable in other organisms. Abundant *let-7* expression in the human brain tissue appears to support this possibility.
